# Gas-dependent plasma activation of a manganese MOF precatalyst tunes defect accessibility and reconstruction under alkaline HER conditions

**DOI:** 10.1039/d6ma00128a

**Published:** 2026-06-08

**Authors:** Constantin Eisen, Anna Strijevskaya, Monnaya Chalermnon, Youven Benseghir, Kevin Doppelmayer, Steven van Terwingen, Marc Pignitter, Michael R. Reithofer, Jia Min Chin

**Affiliations:** a Department of Functional Materials and Catalysis, Faculty of Chemistry, University of Vienna Währinger Straße 42 1090 Vienna Austria jiamin.chin@univie.ac.at; b Institute of Physiological Chemistry, University of Vienna Josef-Holaubek-Platz 2 (UZA II) 1090 Wien Austria; c Institute of Inorganic Chemistry, Faculty of Chemistry, University of Vienna Währinger Straße 42 1090 Vienna Austria michael.reithofer@univie.ac.at; d Wolfgang Pauli Institute Oskar-Morgenstern-Platz 1 1090 Wien Austria

## Abstract

Plasma activation is a practical route to introduce defects in MOF-based electrocatalyst precursors, yet how different plasma environments influence defect formation and subsequent oxide evolution remains unclear. Here, manganese (Mn)-MOFs grown on nickel foam (NF) were treated with Ar, N_2_, and O_2_ glow-discharge plasmas to establish how plasma chemistry governs MOF modification and HER performance. Ar plasma generates a higher population of accessible undercoordinated Mn sites with minimal chemical passivation, which correlates with a more favorable electrochemical reconstruction into a hydrated, layered Mn oxide phase under HER conditions. In contrast, N_2_ and O_2_ plasmas produce additional chemical alterations such as partial nitriding or oxidative linker damage that limited vacancy accessibility and suppressed oxide formation. Comprehensive characterization (PXRD, Raman, XPS, TGA, EPR, SEM) shows that these gas-dependent pathways correlate with the observed catalytic behaviour, with Ar-treated Mn-MOF/NF achieving the lowest overpotential (219 mV at 10 mA cm^−2^) and smallest charge-transfer resistance. These results provide a practical comparison of plasma treatments for activating Mn-MOF electrocatalyst precursors and highlight Ar plasma as an effective route to generate accessible defect sites for MOF catalysis.

## Abstract 2

Gas-dependent plasma activation tunes defects and reconstruction in Mn-MOF precatalysts for alkaline HER. Ar plasma creates accessible Mn sites that promote active Mn oxide formation and deliver the greatest catalytic improvement.

## Graphical abstract

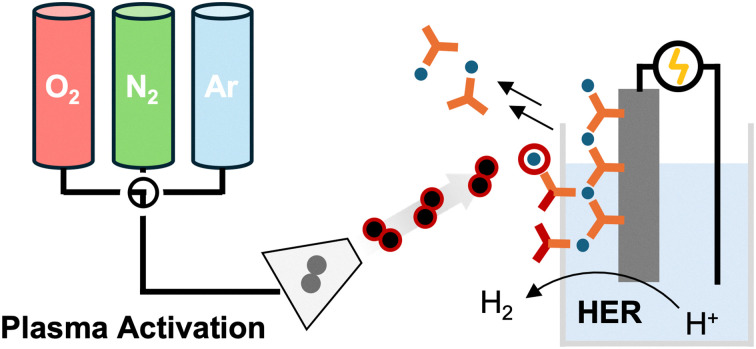

The hydrogen evolution reaction (HER), serving as the cathodic half-reaction in water-splitting, remains central for sustaining a hydrogen-based energy economy.^1^ While noble metals like ruthenium and platinum are considered benchmark catalysts due to their exceptional performance, their scarcity and cost motivate continued exploration of more sustainable, earth-abundant alternatives.^2^ In this context, manganese (Mn)-based materials are particularly attractive due to their accessible and relatively stable redox states under HER conditions^3^ and overall promising electrochemical performance,^4^ as demonstrated in Mn oxides (MnO_*x*_) and bimetallic systems.^5,6^ In comparison, Mn-based metal–organic frameworks (MOFs) remain far less explored for electrocatalysis. Their modular structures offer opportunities for controlled defect generation and precursor engineering,^7^ yet many Mn-MOFs exhibit low surface area and structural rearrangements that limit intrinsic activity.^8,9^

Plasma-aided modification has emerged as a versatile strategy to introduce vacancies, alter linker environments, or partially convert MOFs into catalytically relevant metal oxides to enhance catalytic activity.^10,11^ Most studies focus on plasma treatment using a single gas, typically argon (Ar),^12,13^ nitrogen (N_2_)^14^ or oxygen (O_2_) (Fig. 1).^15^ However, how different plasma chemistries influence defect formation and structural modification of a single MOF-based catalyst has not been systematically explored.

**Fig. 1:**
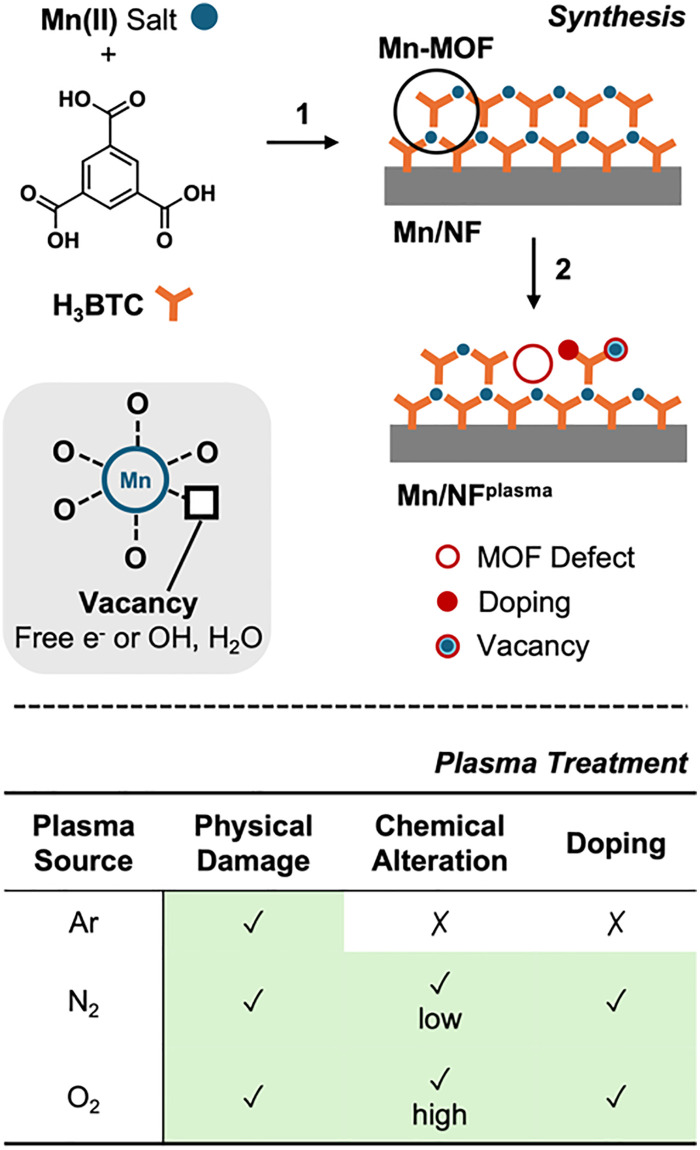
Synthesis of Mn-MOF on NF: (1) – DMF/EtOH/H_2_O, 180 °C, 10 h; (2) – plasma treatment and relevant effects of different plasma source gases.

These gases differ fundamentally in their interaction with the framework: inert Ar plasma mainly induces physical sputtering and bond cleavage, promoting the formation of undercoordinated sites without doping, whereas N_2_ and O_2_ plasmas introduce reactive species that enable N/O incorporation and simultaneous chemical restructuring, which can partially passivate defects (Fig. 1).

In this work, we investigate the gas-dependent activation of Mn-MOFs grown on Ni foam by exposure to glow-discharge plasma using different source gases (Ar, N_2_ and O_2_). We benchmark their HER performance and employ comprehensive structural and spectroscopic characterization (PXRD, Raman, XPS, EPR, and SEM microscopy) before and after catalysis. This comparative approach reveals distinct plasma-specific defect chemistries and transformation pathways, including controlled formation of birnessite-type Mn oxides that govern the observed catalytic behaviour.

## Results and discussion

### Mn/NF synthesis, plasma-treatment and characterization

Mn MOF structures on nickel foam (NF) were obtained by an adapted *in situ* solvothermal MOF growth on NF substrates using manganese(ii) nitrate tetrahydrate, trimesic acid (H_3_BTC) and NF.^16^ Subsequently, timed plasma treatment with argon (Ar), nitrogen (N_2_) and oxygen (O_2_) led to the activated MOF-based catalysts Mn^Ar^/NF, Mn^N2^/NF and Mn^O2^/NF (Fig. 1 and Table 1).

**Table 1:** Comparison of characterization results of Mn-MOF and plasma-treated counterparts

	FE-SEM morphology	PXRD pattern	Raman MnO_*x*_ signals
Mn-MOF	Fine needles	Key peaks are identical	Mn–O lattice ∼420 cm^−1^
Mn-MOF^Ar^			
Mn-MOF^N2^			
Mn-MOF^O2^	Fused needles		Mn(iv)–O 640 cm^−1^

**Fig. 2:**
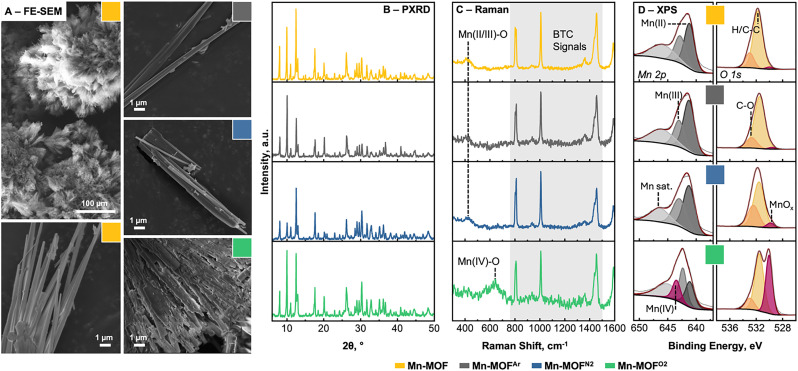
(A) FE-SEM micrographs of Mn-MOFs; (B) PXRD, (C) Raman and (D) high-resolution XPS of Mn 2p and O 1s regions and (D) high-resolution XPS (Mn 2p_3/2_ and O 1s) comparison of Mn-MOF and plasma treated counterparts.

Upon synthesis and plasma treatment, all NF-bound MOFs were characterized by field emission scanning electron microscopy (FE-SEM) to study their morphologies and energy-dispersive X-ray (EDX) maps to confirm their elemental composition. Untreated Mn-MOF and plasma-treated counterparts were carefully removed from the NF surface, and direct micrograph comparison shows that Mn-MOF^Ar^ and Mn-MOF^N2^ retained the needle-like morphology of the untreated Mn-MOF, while Mn-MOF^O2^ shows drastic changes in the crystal morphology. Needle-like crystals Mn-MOF^O2^ partially fuse together, and their edge geometries become rounded (Fig. 2D). EDX maps (see SI, Fig. S6–S8) of all plasma-treated Mn-MOFs show the expected composition, with C/O associated to the BTC linker and Mn/Ni contributions associated with the metal nodes and the support structure, respectively. Despite the morphological differences observed by FE-SEM, all plasma-treated MOFs showed sharp peaks in PXRD, confirming the retention of crystallinity throughout the plasma treatment. PXRD patterns show contributions of reported Mn-BTC coordination polymer^17^ and nanoparticles^18^ (see SI, Fig. S1). Since PXRD data do not allow direct identification of the obtained structure, three-dimensional electron diffraction (3D ED) was employed to gain further structural insights into untreated Mn-MOF through the identification of unit cell parameters. Details are discussed in the supplementary information (see SI, Fig. S2).

**Fig. 3:**
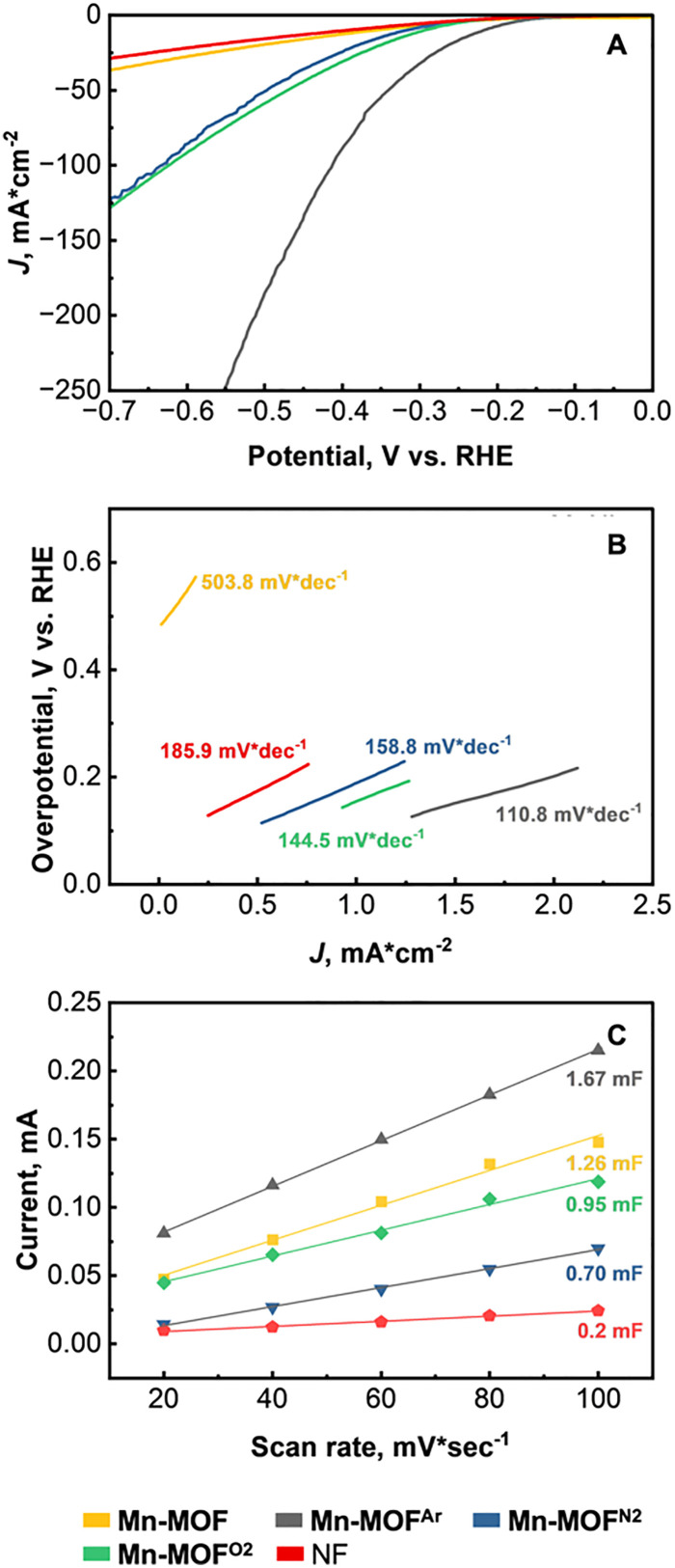
(A) Cathodic polarization curves (LSV), (B) Tafel plots and (C) electrochemical double layer capacitance *C*_dl_ of Mn-MOFs benchmarked against blank NF.

Raman spectroscopy was also carried out to elucidate the type of oxide and organic components present after plasma treatment. Raman spectra of Mn-MOF^N2^ and Mn-MOF^Ar^ show full retention of organic components and oxide contributions at 420 and 558 cm^−1^, associated with Mn–O lattice vibrations^19^ and bending motifs of MnOOH species.^20^ In contrast, Mn-MOF^O2^ shows a broad peak at 640 cm^−1^ associated with Mn(iv)–O asymmetric stretching, indicating the formation of α-MnO_2_.^21^

To confirm oxidation states of Mn and further insights in Mn-MOFs on NF, X-ray photoelectron spectroscopy (XPS) was performed (Table 2; for spectra see SI, Fig. S3 and S4).

**Table 2:** List of XPS contributions of Mn-MOF and plasma-treated counterparts. All values in eV and referenced to C–C/H contribution at 284.8 eV

	Mn 2p	Ni 2p	C 1s	O 1s	N 1s
Mn-MOF	Mn(ii) ∼641	Ni(0) ∼853	C–C/H 284.8	MnO_*x*_ ∼530	N_2_(ads)/DMF ∼400
Mn-MOF^Ar^	Mn(iii) ∼642	Ni(ii) ∼855	C=O ∼286	Mn–OOC ∼531	N_2_(ads)/DMF N–O 402
Mn-MOF^N2^		Ni(iii) ∼856	C–O ∼288	O–C ∼533	N_2_/DMF & N-O
Mn-MOF^O2^	Mn(ii) & Mn (iii)				NO_*x*_ ∼406
	Mn(iv) ∼643				

Ni 2p_3/2_ spectra are essentially identical for all plasma-treated samples and show contributions from metallic Ni and Ni(ii/iii), originating from the underlying nickel foam used for *in situ* growth of the Mn-MOF layer.^16,22,23^ The Mn 2p_3/2_ region of Mn-MOF, Mn-MOF^Ar^ and Mn-MOF^N2^, exhibits two main components at ∼641 eV and ∼642 eV, assigned to Mn(ii) and Mn(iii), respectively (Fig. 2, XPS-Mn 2p).^24^

In contrast, O_2_-plasma treated Mn-MOF^O2^ shows a decreased Mn(ii) satellite at 647–645 eV together with an additional Mn(iv) contribution at ∼643 eV attributed to Mn(iv), in line with the Raman signatures of α-MnO_2_. The C 1s spectra are consistent across all Mn-MOF samples (see SI, Fig. S4), whereas the N 1s and O 1s regions display clear plasma-composition-dependent changes.

N 1s spectra of Mn-MOF and Mn-MOF^Ar^ are dominated by a peak at 400 eV attributed to adsorbed N_2_ and/or residual DMF within the MOF. Mn-MOF^N2^ shows a similar peak at 400 eV with increased relative intensity, together with a minor contribution at ∼402 eV assigned to newly formed N–O bonds during partial nitriding of O-containing moieties under N_2_ plasma. Mn-MOF^O2^ also shows contributions at 400 and 402 eV, attributed to the same species as in Mn-MOF^N2^, but additionally exhibits a pronounced peak at ∼406 eV associated with highly oxidized N species such as NO_2_ groups arising from oxidation of DMF residue or adsorbed N_2_ during more reactive O_2_ plasma treatment.

The O 1s spectra are nearly identical for Mn-MOF, Mn-MOF^Ar^ and Mn-MOF^N2^, with a broad peak at ∼532 eV corresponding to organic C–O/COO and Mn–COO environment, consistent with the carboxylate groups of the BTC linker.^25^ In Mn-MOF^O2^, the contribution at 530–529 eV is drastically reduced, corroborating the formation of Mn oxide phases,^26^ consistent with Raman spectroscopy and Mn 2p_3/2_ XPS contributions.

**Fig. 4:**
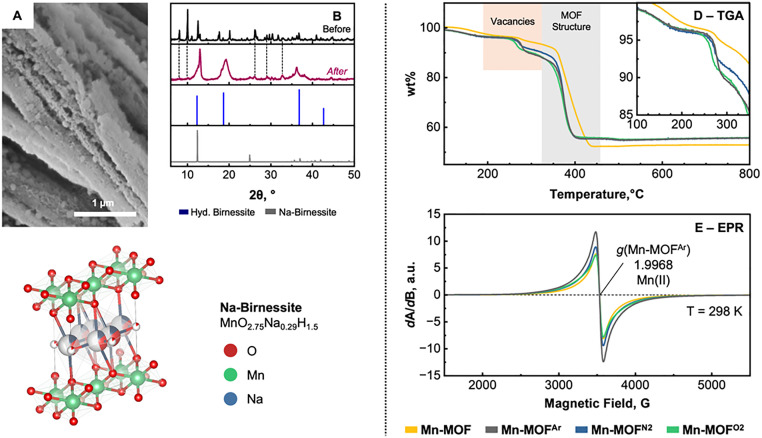
(A) FE-SEM post-HER of Mn-MOF^Ar^; (B) and (C) PXRD comparison pre- and post-HER of Mn-MOF^Ar^ including simulated and diffractograms structure of birnessite; (D) TGA of Mn-MOF and all plasma treated counterparts; (E) EPR of Mn-MOF and all plasma treated counterparts.

Further, XPS determination of carbon/metal ratios shows that all plasma-treated MOFs underwent significant etching of organic components (see SI, Table S1). This was verified by thermogravimetric analysis (TGA; Fig. 4D) which revealed ∼3.5 wt% lower organic contributions for plasma-treated Mn-MOFs relative to the pristine material. The TGA profiles of plasma-treated samples show a two-step degradation process after 200 °C. The first mass loss event at 267 °C is attributed to the removal of labile species such as coordinated H_2_O and hydroxyl groups associated with undercoordinated Mn sites.^27,28^ The second thermal decomposition step is observed at 333 °C, associated with Mn-BTC framework decomposition.^27–31^ Detailed investigation of first decomposition step allows a relative estimation of vacancy depending on the employed plasma-treatment. In Mn-MOF^Ar^, the initial decomposition step (∼267 °C; Fig. 4C) shows a weight loss of ∼6 wt%, assigned primarily to the release of coordinated OH^−^ and H_2_O.^27,28^ Given that TGA was conducted in air, overlapping processes such as partial oxidation cannot be excluded. Therefore, this weight loss is interpreted as a relative indicator of increased vacancies rather than a direct quantitative measure of coordination vacancies. Notably, this value is significantly higher than that of plasma-untreated Mn-MOF.

Additional evidence for the higher vacancy concentration in plasma-treated Mn-MOF^Ar^ is provided by electron paramagnetic resonance (EPR) measurements of Mn-MOF and all plasma-treated analogues. The observed signal (Fig. 4E) arises from EPR-active high-spin Mn(ii) centres (*g* ≈ 2.00), in agreement with previous reports on Mn-BTC MOFs.^31^ All Mn-MOF samples exhibit a broad EPR resonance without resolved hyperfine splitting, reflecting the structural complexity of the framework arising from mixed Mn oxidation states and BTC linkers acting as bridging carboxylates that connect multiple Mn centres.^32,33^ In this environment, Mn(ii) nodes bearing a higher density of coordination vacancies and/or fewer closely bound MOF linkers give rise to increased signal intensity. Amongst the MOF samples, Mn-MOF^Ar^ displays the most intense EPR signal, supporting a higher proportion of undercoordinated Mn(ii) sites in the Ar plasma-treated material.^3,30^

### HER activity of plasma treated Mn/NF

The electrochemical activity for hydrogen evolution reaction (HER) of Mn/NF, Mn^Ar^/NF, Mn^N2^/NF, and Mn^O2^/NF was systematically investigated to compare the impacts of the different plasma-treatments. Experiments were conducted in a standard three-electrode setup, utilizing a 1 M NaOH aqueous solution, purged with argon, as the electrolyte. The as-prepared Mn/NFs, with a geometrical area of 1 cm^2^, functioned as the working electrode, while a Pt mesh served as the counter electrode and an Ag/AgCl electrode (sat. KCl) as the reference. All polarization curves are presented without *iR* compensation. Prior to all electrochemical measurements, each sample underwent a pre-treatment *via* cyclic voltammetry (CV) from 1.3 V *vs.* RHE to −0.7 V *vs.* RHE at a scan rate of 100 mV sec^−1^ until a stable curve was achieved. Faradaic efficiency was not quantified in this study; accordingly, the electrochemical analysis focuses on comparative HER activity trends under identical experimental conditions.

Upon stabilization of Mn/NFs by CV pre-treatment, the electrochemical surface area (ECSA) was determined based on the double-layer capacitance (*C*_dl_; Fig. 3C). Obtained ECSA results show the highest *C*_dl_ value for Mn^Ar^/NF, followed by Mn^O2^/NF and Mn^N2^/NF with 1.67 mF/41.75 cm^2^, 0.95 mF/23.6 cm^2^ and 0.70 mF/17.5 cm^2^ (see SI, Fig. 3C). Comparing these plasma-treated MOFs on NF with untreated Mn/NF (1.26 mF/32.0 cm^2^), only Mn^Ar^/NF outperforms Mn/NF.

The cathodic polarization curves (Fig. 3A) for all samples revealed a significant improvement in HER activity compared to bare NF. Specifically, at a current density of 10 mA cm^−2^ (*η*_10_), the overpotentials were measured as 358 mV for Mn/NF, 317 mV for Mn^N2^/NF, 295 mV for Mn^O2^/NF, and an optimal 219 mV for Mn^Ar^/NF. To decouple the contribution of the underlying NF substrate, bare NF subjected to identical Ar, N_2_, and O_2_ plasma treatments was evaluated (see SI, Fig. S17). While plasma-treated NF exhibits improved HER activity compared to untreated NF, the *η*_10_ values for NF^N2^ (229 mV) and NF^O2^ (245 mV) indicate no enhancement upon incorporation of the corresponding Mn-MOF layer. In contrast, Mn^Ar^/NF significantly outperforms NF^Ar^ (225 mV), demonstrating a substantially lower overpotential and higher current densities. This comparison clearly shows that, although plasma activation of NF contributes to HER activity, only Mn^Ar^/NF exhibits a pronounced additional enhancement, confirming that its superior performance originates from modifications within the Mn-MOF-derived layer rather than substrate effects alone.

Correspondingly, the Tafel slope for Mn/NF was determined to be 503.8 mV dec^−1^, while plasma treatment effectively reduced these values to 158.8 mV dec^−1^ for Mn^N2^/NF, 144.5 mV dec^−1^ for Mn^O2^/NF, and a remarkably low 110.8 mV dec^−1^ for Mn^Ar^/NF. Further, current densities are normalized to the geometric electrode area, while differences in electrochemically accessible surface area are evaluated independently using double-layer capacitance measurements. Additionally, electrochemical impedance spectroscopy (EIS) was conducted. The resulting Nyquist plots are included in the SI (see SI, Fig. S6). The diameter of the semicircle in these plots directly corresponds to the charge transfer resistance (*R*_ct_), while the solution resistance (*R*_sol_) can be estimated from the high-frequency intercept. Mn^Ar^/NF exhibited the smallest *R*_ct_ value of 6.16 Ω, significantly outperforming Mn^N2^/NF (18.26 Ω), Mn^O2^/NF (15.52 Ω), Mn/NF (39.05 Ω), and bare NF (137.75 Ω). The combination of smaller Tafel slopes and reduced charge-transfer resistance is consistent with more favorable charge-transfer kinetics, thereby supporting the superior HER activity observed for the plasma-treated samples, particularly Mn^Ar^/NF.

The measured electrochemical activity correlates well with the ECSA trends, with Mn^Ar^/NF showing the highest activity and the largest ECSA. This indicates that the predominantly physical surface modification induced by Ar plasma generates more accessible catalytic sites.^34,35^ In contrast, N_2_- and O_2_-plasma treatments also increase the ECSA but introduce additional chemical modifications to the MOF surface. These treatments generate highly reactive surface centres that subsequently undergo chemical passivation through oxygen incorporation, or N-doping induced structural rearrangements that limits the accessibility of Mn sites. As a result, despite modest ECSA gains, Mn^N2^/NF and Mn^O2^/NF exhibit fewer active catalytic centres and ultimately show lower activity than the untreated Mn/NF precursor.

### Post-HER characterization and structure/property relations

PXRD patterns of Mn-MOF samples collected after HER testing reveal retention of the principal framework reflections, accompanied by changes in relative peak intensities and the emergence of additional low-angle features indicative of structural reconstruction (Fig. 4B).^36^ These additional reflections are consistent with the formation of a hydrated, layered MnO_*x*_ phase commonly associated with birnessite-type structures under alkaline electrochemical conditions.^37^ In particular, the shift of a low-angle reflection from ∼25° to ∼19° can be attributed to interlayer expansion upon hydration, a characteristic feature of layered MnO_*x*_ phases formed during electrochemical cycling in alkaline media (Fig. 4B).^38^ Given the hydrated and partially disordered nature of such reconstructed phases, the assignment is only based on PXRD natures and should be interpreted as indicative of birnessite-type MnO_*x*_ rather than a definitive identification of a specific polymorph. Furthermore, no reflections attributable to metallic Mn are observed, suggesting that the formed MnO_*x*_ phase remains stable under the applied conditions.^3^

Among the investigated samples, Mn^Ar^/NF exhibits one of the most pronounced oxide-related reflections following HER operation, consistent with Ar-plasma-induced physical activation generating a higher density of accessible undercoordinated Mn sites that serve as favorable initiation points for electrochemical reconstruction.^34,35^ In contrast, Mn-MOF^N2^ displays weaker oxide signatures, suggesting that reactive plasma-induced chemical modifications limit the extent of reconstruction under identical HER conditions. Mn-MOF^O2^ exhibits similarly distinct oxide-related reflections post-HER as observed for Mn^Ar^/NF. However, the origin of these features differs in terms of the reconstruction pathway. For Mn^O2^/NF, pre-HER characterization (Raman and XPS, Fig. 2C and D) already indicates the presence of MnO_*x*_ species, and SEM micrographs reveal morphological changes induced by O_2_-plasma treatment. This suggests that oxide formation is initiated prior to electrochemical operation. As a result, the extent of additional transformation during HER is likely limited compared to Mn^Ar^/NF, where oxide formation predominantly occurs *in situ* under electrochemical conditions. This distinction in the timing of oxide formation provides a consistent explanation for the comparable PXRD signatures observed after HER, despite fundamentally different underlying reconstruction processes.

Post-HER FE-SEM micrographs further support these trends. While the general MOF crystal shape was retained in the case of Mn-MOF and Mn-MOF^Ar^, Mn-MOF^N2^ and Mn-MOF^O2^ undergo substantial morphological transformation into irregular, polydisperse sheet-like structures (see SI, Fig. S11). The best performing catalyst, Mn-MOF^Ar^ also shows the most significant nanoscale roughening of the MOF crystals. Such formed nanostructures not only enhance HER performance, but act as initiation points for the sacrificial conversion of the MOF into catalytic Mn oxides. The resulting nanostructured Mn oxide increases the availability of electrochemically accessible Mn sites, as reflected by highest ECSA observed for Mn-MOF^Ar^. Post-HER EDX maps (see SI, Fig. S12–S15) reveal increase presence of O, consistent with the formation of MnO_*x*_. Continuous presence of C can be associated with remaining Mn-MOF fragments underneath the MnO_*x*_ layer.

## Summary

In summary, Ar-based glow-discharge plasma treatment is the most effective activation strategy for Mn-MOF/NF, leading to enhanced HER activity in alkaline electrolyte. Ar-treatment increases the electrochemically accessible surface area and the density of vacant coordination sites, corresponding to improved catalytic activity. In contrast, N_2_ and O_2_ plasmas introduce chemically reactive species that drive surface oxidation, N-doping, and other rearrangements, thereby passivating Mn centers and suppressing the formation of catalytically active defects.

As a result, Mn^Ar^/NF displays the best HER activity, with an overpotential of 219 mV at 10 mA cm^−2^, favorable kinetics based on obtained Tafel slopes (110.8 mV dec^−1^) and low charge transfer resistance (6.16 Ω). The improved activity is consistent with the increased ECSA (41.75 cm^2^). Further, control experiments of NF treated with Ar plasma are showing the clear benefit of Mn-MOF^Ar^ over direct treatment of NF.

Post-catalysis structural analysis reveals that Ar plasma not only introduces active vacancies, but also predisposes the MOF to undergo controlled, sacrificial transformation into a nano-textured layer of an active Mn oxide phase in the form of hydrated birnessite. This highlights the dual role of Ar-plasma induction of defects, which not only initial HER activity but also steers the MOF toward an advantageous precatalyst-to-oxide conversion pathway.^39^ Beyond advancing Mn-based HER catalysts, the plasma-activation approach established here offers a general, scalable way to tune MOF electrocatalysts, particularly in systems where limited accessibility to active metal sites constrains performance.

## Author contributions

A.S. and C.E. designed and performed the synthesis and validation experiments. Y.B. wrote the initial draft and performed BET and PXRD analyses. K.D. and M.P. carried out the EPR measurements. S.v.T. performed 3D ED analyses. M.C. conducted electrochemical measurements on reference materials. J.M.C. and M.R.R. acquired funding and supervised the study. C.E., J.M.C., and M.R.R. wrote the manuscript. All authors have approved the final version of the manuscript.

## Conflicts of interest

There are no conflicts to declare.

## Supplementary Material

MA-007-D6MA00128A-s001

## Data Availability

The data supporting this article have been included as part of the supplementary information (SI). Supplementary information: additional data sets including PXRD, XPS, FE-SEM, EDX maps as well as electrochemical data sets including EIS and reference LSVs are included in the SI. SI also contains a brief discussion on the structure of Mn-MOF based on all gathered data sets. See DOI: https://doi.org/10.1039/d6ma00128a.
