# Supplementary material for: Gas-dependent plasma activation of a manganese MOF precatalyst tunes defect accessibility and reconstruction under alkaline HER conditions
Source: Mater Adv. 2026 Jun 8;7(14):7307–13. doi: 10.1039/d6ma00128a (PMC13321717; doi:10.1039/d6ma00128a)
Supplement: MA-007-D6MA00128A-s001 [file MA-007-D6MA00128A-s001.pdf]

Supplementary Information (SI) for Materials Advances.
This journal is © The Royal Society of Chemistry 2026

Electronic Supporting Information

Gas-Dependent Plasma Activation of a Manganese MOF Precatalyst Tunes Defect Accessibility and Reconstruction under Alkaline HER Conditions

Constantin Eisen[a], Anna Strijevskaya[a], Monnaya Chalermnon[c], Youven Benseghir[a], Kevin Doppelmayer[b], Steven van Terwingen[c], Marc Pignitter[b], Michael R. Reithofer[c*], Jia Min Chin[a*]

[a] Department of Functional Materials and Catalysis, Faculty of Chemistry, University of Vienna, Währinger Straße 42, 1090 Vienna, Austria
E-mail: jiamin.chin@univie.ac.at
[b] Institute of Physiological Chemistry, University of Vienna, Josef-Holaubek-Platz 2 (UZA II), 1090 Wien, Austria
[c] Institute of Inorganic Chemistry, Faculty of Chemistry, University of Vienna, Währinger Straße 42, 1090 Vienna, Austria
E-mail: michael.reithofer@univie.ac.at

Table of Contents

Materials and Methods 3
Synthesis of Mn/NF 4
Plasma Treatment 4
Electrochemical Hydrogen Evolution 4
PXRD Comparison 5
MOF Structure 6
High-resolution XPS Spectra 7
XPS-based C/Mn 8
Electrochemistry Plots – LSV with Pt Reference and EIS 9
EDX Maps of Mn-MOFs 9
FE-SEM after HER 11
EDX Maps of Mn-MOFs after HER 12
PXRD after HER 13
Cathodic Polarization Curves of NF 13
References 14

Materials and Methods

All experiments, if not stated otherwise, were performed under ambient conditions. Commercially available reagents were used without further purification. Nickel foam (NF, 99.5%, 0.45 g cm^{-3}, thickness 1.6 mm) was obtained from Goodfellow. Ultra-pure water (18.2 MΩ.cm) was obtained by Mili-q water purification system.

Low Pressure Glow Discharge Plasma experiments were performed on a Plasma Diener Atto System (0−300 W / 13.56 MHz) at a power of 150 W and a reduced pressure of 0.4 mbar. Plasma source gasses (Ar, N_2 and O_2) were obtained by Messer Schweiz AG at a purity of 99.999%.

Field Emission Scanning electron microscope (FE-SEM) images of the MOF electrodes were captured on a Zeiss Supra 55 VP electron microscope, coupled with Oxford instruments EDX X-max 80 mm2 detector at 20 kV.

Field emission scanning electron microscopy (FE-SEM) was performed on a Zeiss Supra 55 VP electron microscope, coupled with an Oxford Instruments *energy-dispersive X-Ray* (EDX) EDX-X-max 80 mm^2 detector at 20 kV at Faculty Centre for Nano Structure Research, Faculty of Chemistry/Physics, University of Vienna.

Powder X-Ray diffraction (PXRD) measurements were carried out on an Empyrean Panalytical. The anode material is Cu, Step Size [°2θ] is 0.0130, Generator Settings: 40 mA, 45 kV, measurement was performed at ambient temperature.

Confocal Raman spectra were acquired using a WITec alpha300 A confocal Raman microscope equipped with a 785 nm laser, and a high-throughput spectrometer coupled to a cooled CCD detector (DU401_Br, DD). Measurements were performed using a Zeiss LD EC Epiplan-Neofluar 100x / 0.9. The laser power at the sample was set to a maximum of 25 mW. All spectra were processed using WITec Project software (version 7.0) and plotted with Origin Pro v9.7.5.184.

X-ray photoelectron spectroscopy (XPS) was performed on a Nexsa Photoelectron Spectrometer (Thermo Fisher Scientific, UK) provide by the Core Facility "Interface Characterization", Faculty of Chemistry, University of Vienna. Powder samples were drop casted as suspensions on pre-cleaned silicon wafers and dried for 24 h at 60 °C. Element specific high-resolution spectra for carbon (C 1s 279-298 eV), nitrogen (N 1s 392-410 eV), oxygen (525-545 eV), manganese (630-665 eV) and nickel (844-890 eV) were recorded with step size of 0.1 eV and a pass energy of 50 eV using 30 scans. All measurements were performed using Al-Kα X-rays with a spot size of 400 µm. Obtained spectra were evaluated using the Advantage software package v5.9929 provided by Thermo Fisher Scientific and plotted with Origin Pro v9.7.5.184.

Thermogravimetric analysis (TGA) was performed on a Netzsch STA 449 F3 Jupiter under air (from a gas bottle, mix = 80/20). Samples were heated to 110 °C at a rate of 10 $°C \cdot min^{-1}$ and held at this temperature for 15 min. Following the isothermal step, samples were heated to 700 °C at a rate of 10 $°C \cdot min^{-1}$.

Electron Paramagnetic Resonance (EPR) measurements were taken on a X-band Bruker Elexsys-II E500 ESR spectrometer (Bruker Biospin GmbH). Experiments were conducted at a microwave frequency of 9.851 GHz with a modulation frequency of 100 kHz, sweep time was 10.3 seconds, and a sweep width of 4000 G. The modulation amplitude was set to 19.590 G and the microwave power was 0.6325 mW. The centre field was fixed at 3500 G with a resolution of 1024 points along the x-axis. All measurements were performed using 100 µL capillaries loaded with sample (0.004 g).

3D Electron Diffraction (ED) data were collected on a JEM2100+ (JEOL Ltd., Tokyo, Japan) transmission electron microscope (TEM) with a LaB6 cathode (Denka Ltd., Tokyo, Japan) electron source. A 1024x1024 pixel JUNGFRAU detector with a 320 µm Si sensor and a pixel size of 75 mm^2 was used. The sample for ED was put onto a grid (Cu, lacey carbon, 200 mesh) by dipping the grid in the powdered sample. A GATAN Elsa model 698 high-tilt cryo holder (GATAN Inc., Pleasanton, USA) was used. The sample was measured at room temperature (22 °C). The data were collected by the rotation method, where in the ideal case 120° (rotation from −60° to 60°) were covered. For some crystals, the amount of rotation had to be limited, e.g. due to the proximity of the crystal to the grid bars. Data was analysed with the XDS software package[1] to derive the crystal's unit cell dimensions.

Synthesis of Mn/NF

Mn-MOF was grown *in situ* on NF support using a solvothermal method. $Mn(NO_3)_2 \cdot 4\ H_2O$ (1.180 g, 4.700 mmol) and trimesic acid (H_3BTC, 0.150 g, 0.300 mmol) were dissolved in DMF/EtOH/H_2O v/v 1:1:1 (30 mL). NF (1x1 cm^2) was added to the solution and sonicated for 15 min. The solution and NF piece was transferred into a Teflon-lined stainless-steel autoclave and heated to 180 °C for 10 h. Upon cooling to RT, the NF piece was rinsed with copious amounts of EtOH and acetone, followed by soaking in boiling EtOH for 1 h. The final **Mn/NF** piece was dried in an oven at 60 °C.

Note: Plasma treated **Mn-MOF** was obtained by careful removal of MOF layer from **Mn/NF** sample by scratching.

Plasma Treatment

Dried **Mn/NF** samples were treated for 4 mins by low pressure glow discharge plasma using argon (Ar), nitrogen (N_2) and oxygen (O_2) as plasma source. During the treatment, the system pressure was kept at 0.4 mbar and power was fixed at 150 W. Obtained samples are denoted **Mn^{Ar}/NF**, **Mn^{N2}/NF**, and **Mn^{O2}/NF**, according to used plasma source.

Electrochemical Hydrogen Evolution

Catalytic activity was evaluated using Autolab PGSTAT302N from Metrohm AG with three-electrode set up and 1 M NaOH aqueous solution (Ar saturated, pH 14, 40 mL) as electrolyte. A silver-silver chloride (Ag/AgCl, sat. KCl) electrode and a platinum mesh (1 cm^2) were used as the reference (RE) and counter electrodes (CE), respectively. All potentials mentioned were converted to RHE using the equation 1.

$$E_{RHE} = E^0 + 0.197 + 0.059 \cdot pH \qquad \text{Eq. 1}$$

The double layer capacitance C_{dl} was estimated from the slope of the linear regression between current density and the scan rate. The specific capacitance C_s of 0.02mFcm^{-2} was used for determination of ECSA. The electrochemically active surface area of the as-prepared electrodes has been calculated using the following equation 2-3.

$$ECSA = \frac{C_{dl}}{C_s} \qquad \text{Eq. 2}$$

Where,

$$C_{dl} = \frac{1}{2} \cdot \Delta J = \frac{1}{2}(J_a - J_c) \qquad \text{Eq. 3}$$

Platinum on Carbon (Pt/C) Reference – Pt/C Ink formulation was prepared by sonication of 4.96 mg of 10wt% Pt/C (Sigma Aldrich) with 700 µL MilliQ water (18.2 MΩ, 25°C), 250 µL Isopropanol, 50 µL 5% Nafion (Ion Power). Ink (3 µL) was applied and dried on a polished Glassy-carbon electrode (GCE, polished with 0.05 µm Alumina). The Pt/C coated GCE was used in the identical RE and CE setup as previously stated at a rotating speed of 1600 rpm.[2]

All polarization curves and Tafel plots were obtained at potential sweeping rate of 5 mVsec^{-1}. No *iR*-drop compensation was applied to all reported electrochemical measurements. *iR* compensation would reduce the absolute overpotential values, the relative activity trends between the differently plasma-treated samples are not expected to change, as all measurements were conducted under identical experimental conditions.

Current densities were normalized by the geometric area of the electrodes unless stated otherwise.

PXRD Comparison

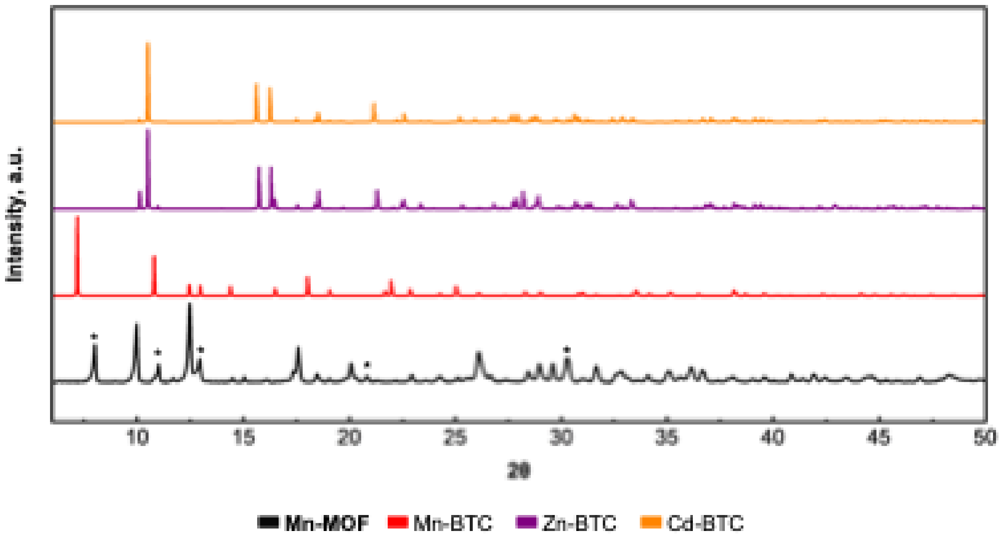

Figure S1 Mn-MOFs grown in this study closely match the Mn-BTC coordination polymer reported by Kitagawa and coworkers[3] and shares key similarities with Mn-BTC nanoparticles of Lin and coworkers.[4] Relevant matching signals with Lin and coworkers are marked (*, major signals ~ 6, 11, 16, 21, 30 °). Zn-BTC and Cd-BTC simulated from literature reports.

MOF Structure

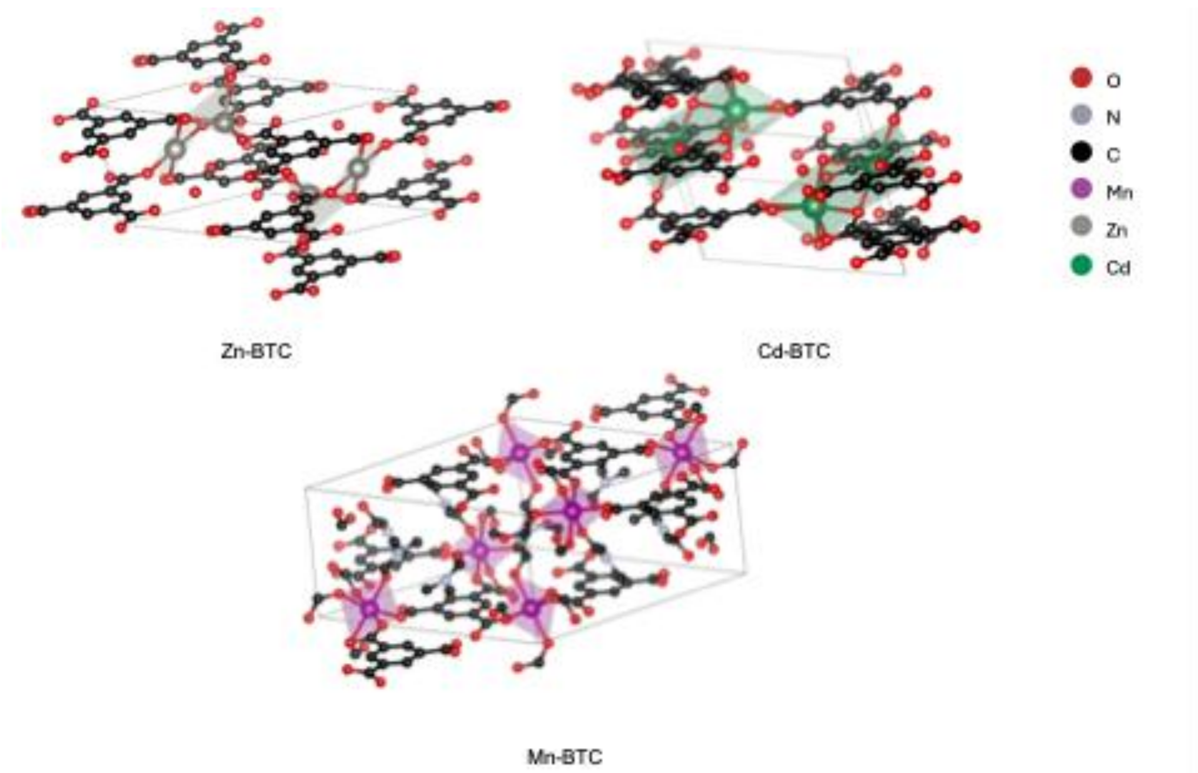

	Space group	a, Å	b, Å	c, Å	α, °	β, °	γ, °
**Mn-MOF** (from *ED*)	P-1 triclinic	7.162(7)	9.861(16)	9.91(2)	64.84(16)	79.33(13)	78.54(9)
Zn-BTC	P-1 triclinic	6.4923(5	9.5272(8)	9.8794(8)	62.829(2)	78.968(2)	78.855(2)
Cd-BTC	P-1	6.5573(10)	9.5294(14)	9.8799(15)	62.979(2)	79.162(2)	78.3850(10)
Mn-BTC	P-3 trigonal	14.2146(10)	14.2146(10)	8.1976(8)	90	90	120

Figure S2 Obtained PXRD patterns of **Mn-MOF** show similarities with previous reports of Mn-BTC MOFs but do not offer a direct match.[3-4] In order to get further insights in the structure of Mn-MOF 3D electrion diffraction (ED) has been employed. Due to poor crystal quality and the low triclinic symmetry only unit cell parameters were established. Matching of unit cell parameters did not resulted in a direct match but led to two structures based on BTC linkers – Zn(II)-BTC[5] and Cd(II)-BTC.[6] Using all gathered analytical data (XPS, TGA, EPR) from Mn-MOF the following structural elements can be assigned: Mn-MOF features a Mn/BTC ratio of ~3:1 based on TGA matching the structure of Kitagawa and coworkers. EPR and XPS data suggest Mn(II) in a octahedral coordination sphere with coordination vacancies occupied by OH or H_2O. Since XPS suggest the presence of Mn(III) distorted octahedral coordination geometries based on the Jahn-Teller effect can be expected leading to more complex structures as reported for the Zn/Cd based systems with matching unit cell parameters.[7] Further, XPS suggest residual DMF or decomposition products of DMF embedded in the structure adding additional layer of complexity.

High-resolution XPS Spectra

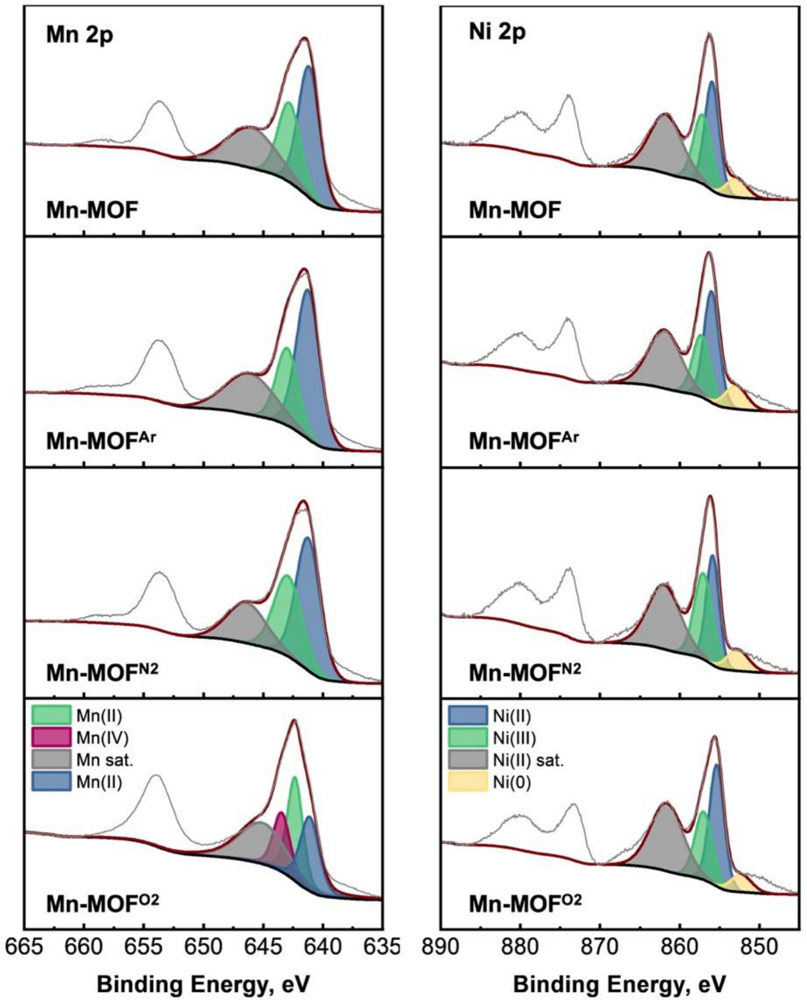

Figure S3 High-resolution XPS spectra in Mn 2p and Ni 2p region of **Mn-MOF** and plasma-treated counterparts.

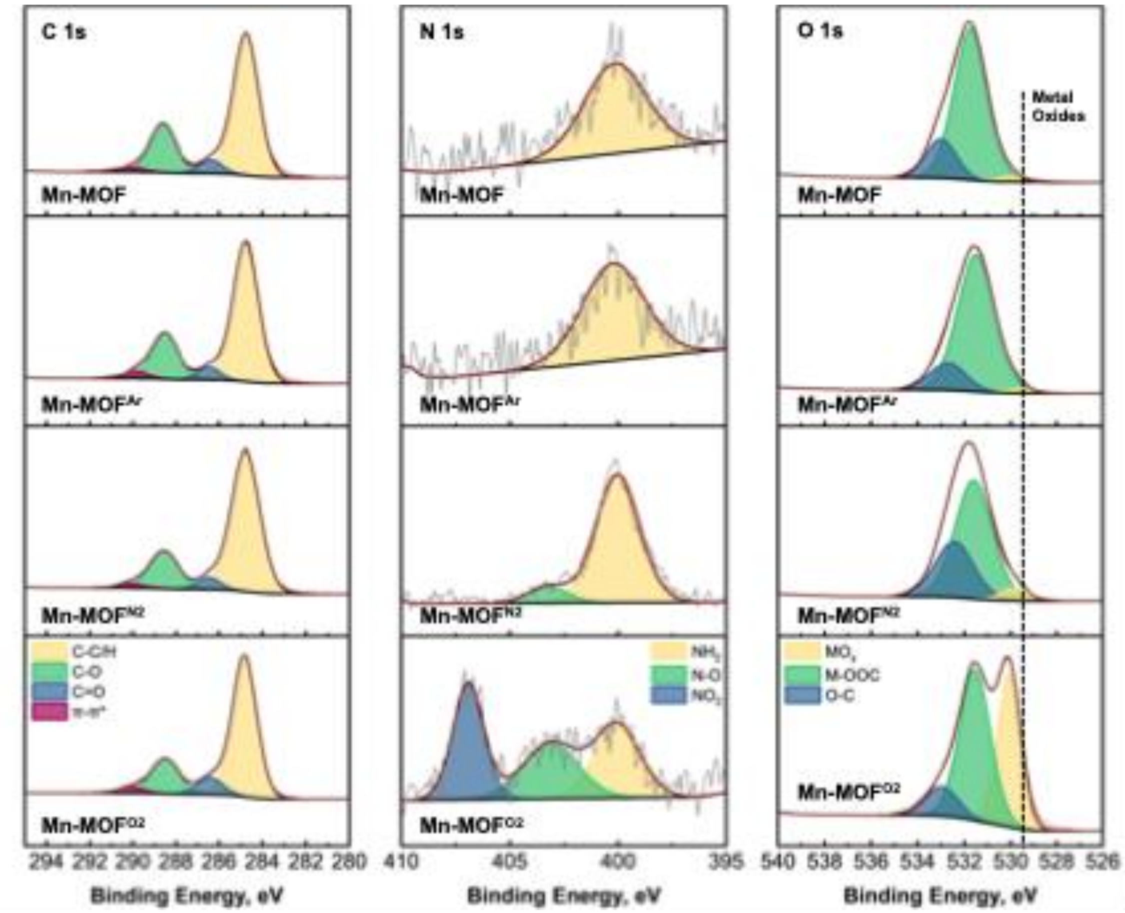

Figure S4 High-resolution XPS spectra in C 1s, N 1s and O 1s region of **Mn-MOF** and plasma-treated counterparts. Reference line for MO_x (530-529 eV) based on Biesinger *et al.*[8]

XPS-based C/Mn

Table S1 Molar ratios of C/Mn region based on XPS survey.

	C/Mn, molar ratio
**Mn-MOF**	8.2 : 1
**Mn-MOF^{Ar}**	4.1 : 1
**Mn-MOF^{N2}**	6.1 : 1
**Mn-MOF^{O2}**	2.2 : 1

Electrochemistry Plots – LSV with Pt Reference and EIS

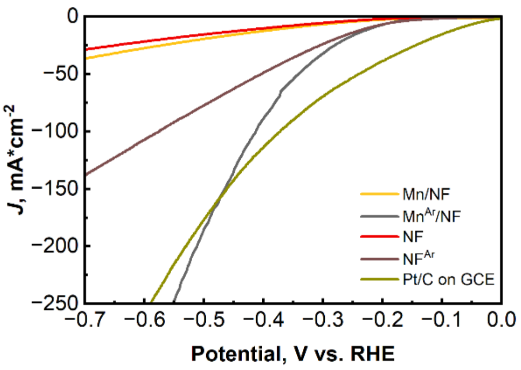

Figure S5 LSV curves of **Mn/NF** and NF versus Pt/C on GCE (0.2 mg cm^{-2}, 1600 rpm).

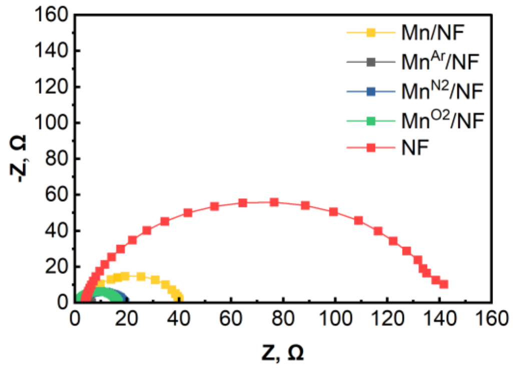

Figure S6 EIS spectra of **Mn/NF** and plasma-treated counterparts.

EDX Maps of Mn-MOFs

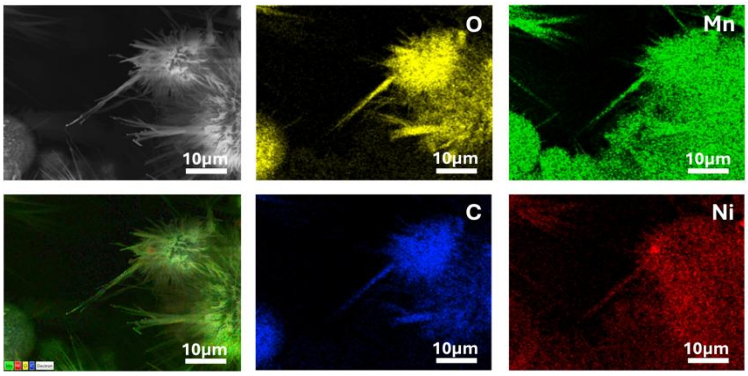

Figure S7 EDX elemental maps (O, Mn, C, Ni) of **Mn-MOF**.

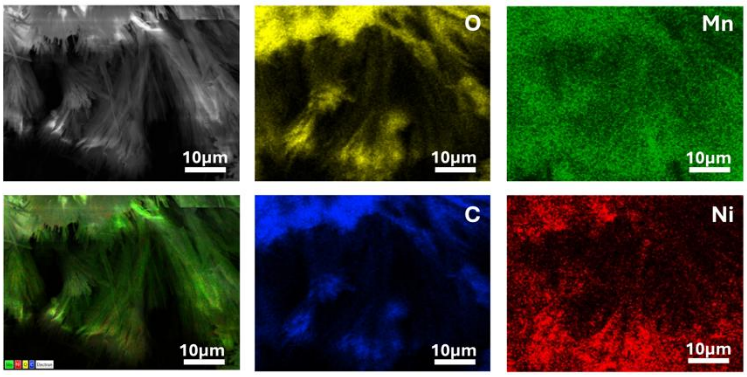

Figure S8 EDX elemental maps (O, Mn, C, Ni) of **Mn-MOF^{Ar}**.

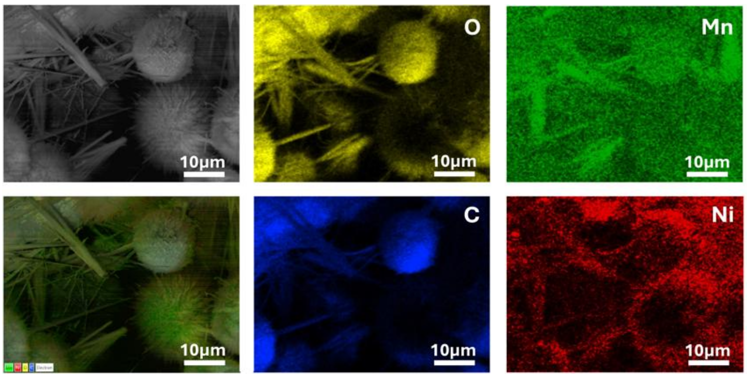

Figure S9 EDX elemental maps (O, Mn, C, Ni) of **Mn-MOF^{N2}**.

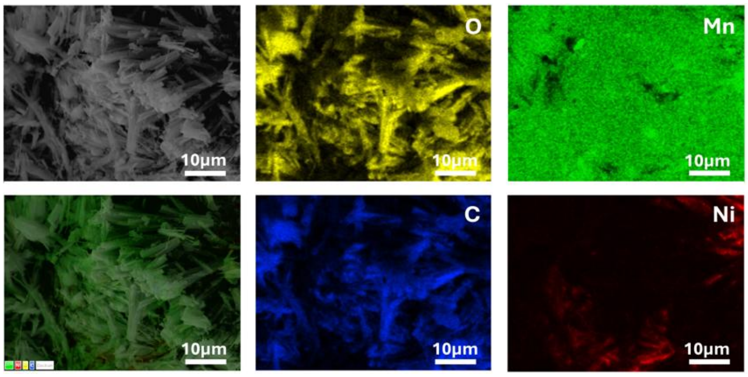

Figure S10 EDX elemental maps (O, Mn, C, Ni) of **Mn-MOF^{O2}**.

FE-SEM after HER

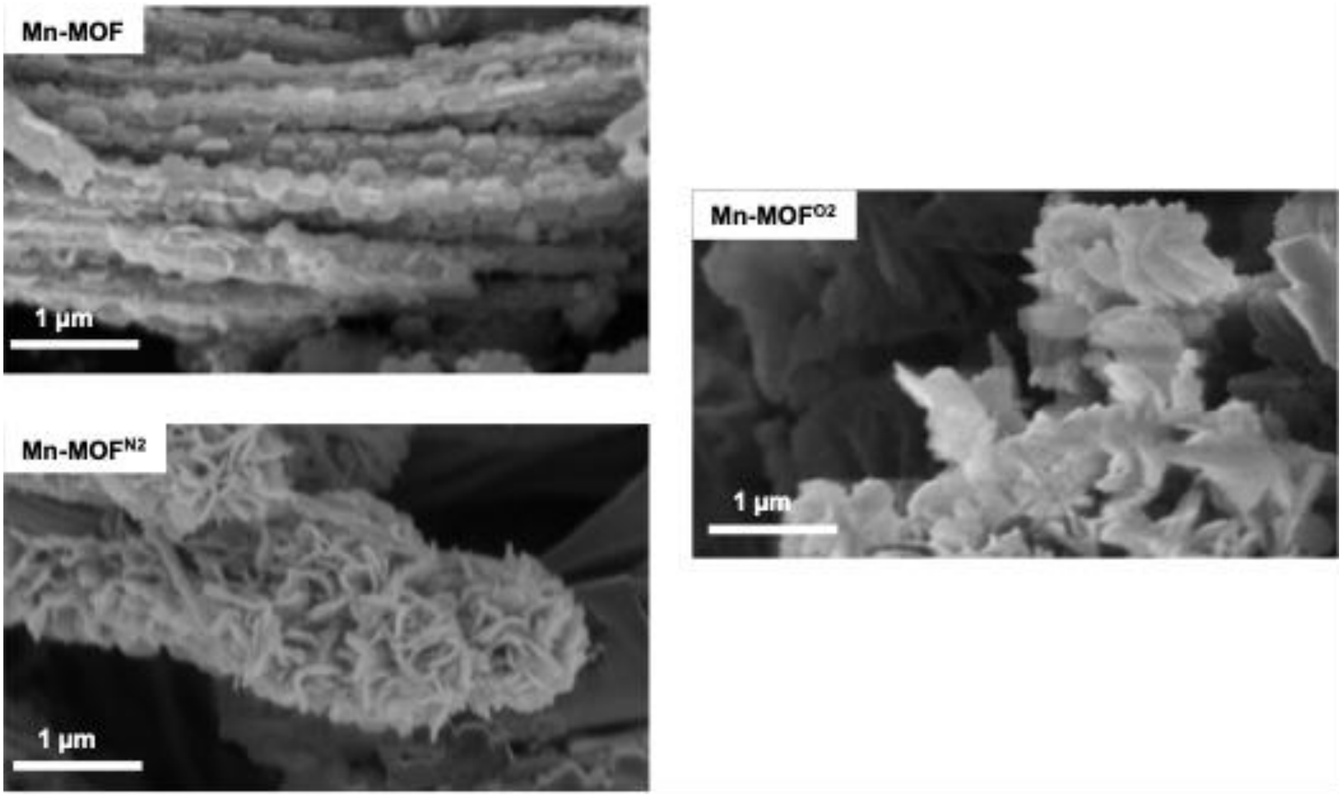

Figure S11 FE-SEM micrographs of **Mn-MOF**, **Mn-MOF^{N2}** and **Mn-MOF^{O2}**.

EDX Maps of Mn-MOFs after HER

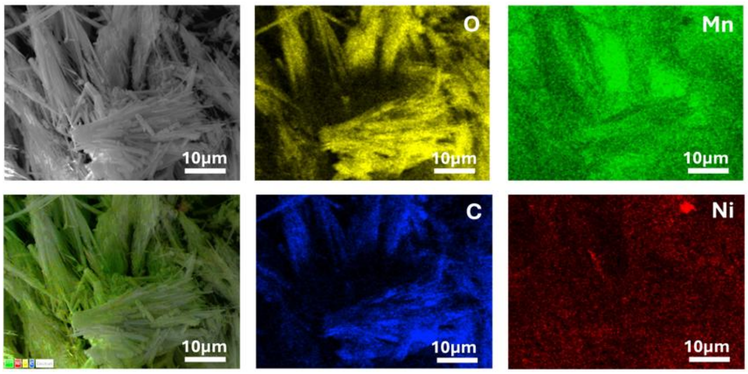

Figure S12 EDX elemental maps (O, Mn, C, Ni) of **Mn-MOF** after HER.

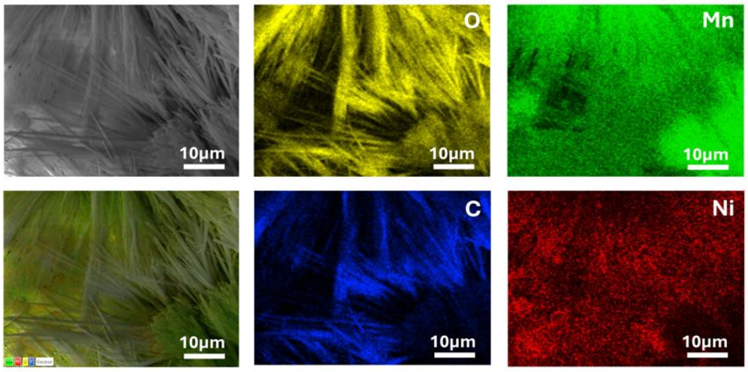

Figure S13 EDX elemental maps (O, Mn, C, Ni) of **Mn-MOF^{Ar}** after HER.

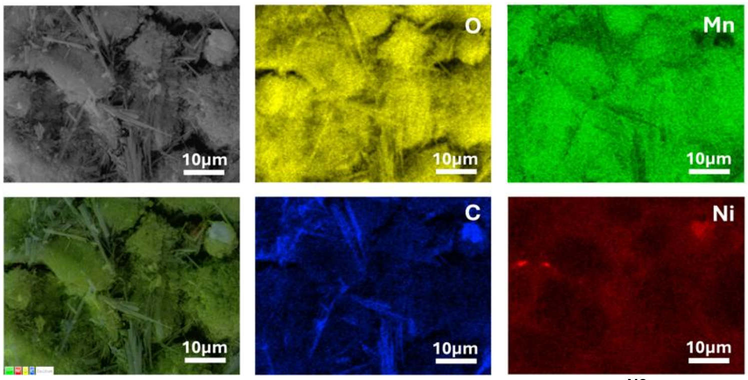

Figure S14 EDX elemental maps (O, Mn, C, Ni) of **Mn-MOF^{N2}** after HER.

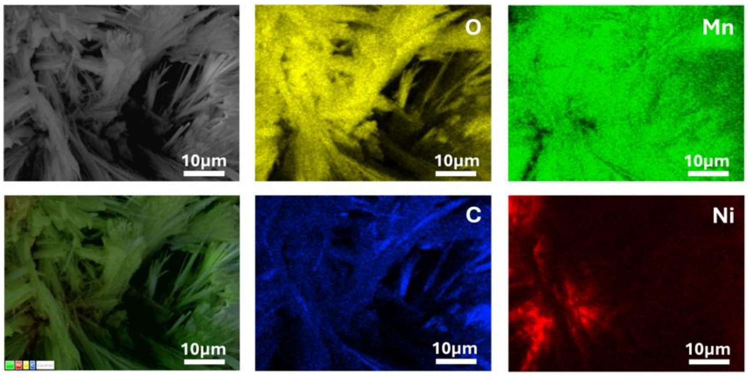

Figure 15 EDX elemental maps (O, Mn, C, Ni) of **Mn-MOF^{O2}** after HER.

PXRD after HER

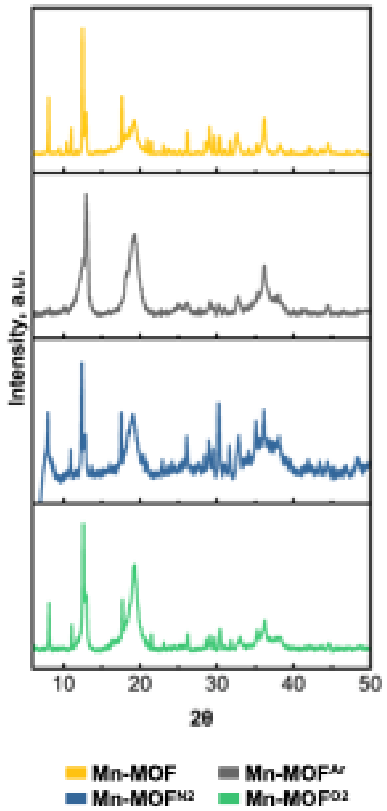

Figure S16 PXRD diffractograms after HER of **Mn-MOF** scratched from NF and plasma-treated counterparts.

Cathodic Polarization Curves of NF

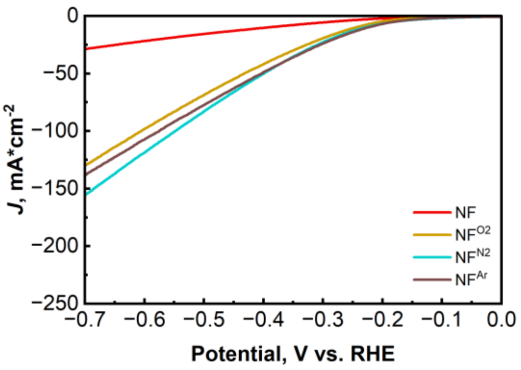

Figure S17 LSV of blank and plasma-treated NF.

References

[1] W. Kabsch, *Acta Crystallogr., Sect. D:Biol. Crystallogr.* **2010**, *66*, 125-132.
[2] Hui Yuan, Jiantao Li, Wei Yang, Zechao Zhuang, Yan Zhao, Liang He, Lin Xu, Xiaobin Liao, Ruiqi Zhu, L. Mai, *ACS Appl. Mater. Interfaces* **2018**, *10*, 16410–16417.
[3] J. Chen, M. Ohba, S. Kitagawa, *Chem. Lett.* **2006**, *35*, 526-527.
[4] K. M. Taylor, W. J. Rieter, W. Lin, *J. Am. Chem. Soc.* **2008**, *130*, 14358-14359.
[5] L. Xu, E. Y. Choi, Y. U. Kwon, *Inorg. Chem.* **2007**, *46*, 10670-10680.
[6] Y.-F. Zhou, B.-L. Wu, L. Han, M.-C. Hong, *Acta Crystallogr., Sect. E:Struct. Rep. Online* **2004**, *61*, m160-m163.
[7] S. Zein, C. Duboc, W. Lubitz, F. Neese, *Inorg. Chem.* **2008**, *47*, 134-142.
[8] M. C. Biesinger, B. P. Payne, A. P. Grosvenor, L. W. M. Lau, A. R. Gerson, R. S. C. Smart, *Appl. Surf. Sci.* **2011**, *257*, 2717-2730.
